# Measuring Cognitive Errors Using the Cognitive Distortions Scale (CDS): Psychometric Properties in Clinical and Non-Clinical Samples

**DOI:** 10.1371/journal.pone.0105956

**Published:** 2014-08-29

**Authors:** Kadir Özdel, Ibrahim Taymur, Seher Olga Guriz, Riza Gökcer Tulaci, Erkan Kuru, Mehmet Hakan Turkcapar

**Affiliations:** 1 Diskapi Yildirim Beyazit Teaching and Research Hospital, Department of Psychiatry, Ankara, Turkey; 2 Sevket Yilmaz Teaching and Research Hospital, Department of Psychiatry, Bursa, Turkey; 3 Hasan Kalyoncu University, Department of Psychology, Istanbul, Turkey; University of Stellenbosch, South Africa

## Abstract

The Cognitive Distortions Scale was developed to assess thinking errors using case examples in two domains: interpersonal and personal achievement. Although its validity and reliability has been previously demonstrated in non-clinical samples, its psychometric properties and scoring has not yet been evaluated. The aim of the current study was to evaluate the psychometric properties of the Cognitive Distortions Scale in two Turkish samples and to examine the usefulness of the categorical scoring system. A total of 325 individuals (Sample 1 and Sample 2) were enrolled in this study to assess those psychometric properties. Our Sample 1 consisted of 225 individuals working as interns at the Diskapi Yildirim Beyazit Teaching and Research Hospital and Sample 2 consisted of 100 patients diagnosed with depression presenting to the outpatient unit of the same Hospital. Construct validity was assessed using the Beck Depression Inventory, the State Trait Anxiety Inventory, the Dysfunctional Attitude Scale, and the Automatic Thought Questionnaire. Factor analyses supported a one-factor model in these clinical and non-clinical samples. Cronbach's α values were excellent in both the non-clinical and clinical samples (0.933 and 0.918 respectively). Cognitive Distortions Scale scores showed significant correlation with relevant clinical measures. Study Cognitive Distortions Scale scores were stable over a time span of two weeks. This study showed that the Cognitive Distortions Scale is a valid and reliable measure in clinical and non-clinical populations. In addition, it shows that the categorical exists/does not exist scoring system is relevant and could be used in clinical settings.

## Introduction

Beck's cognitive theory has been one of the most influential contributions in the field of psychotherapy [1]. The main proposal of cognitive theory is that the emotional and behavioral reactions of individuals fundamentally depend upon underlying cognitive structures such as beliefs and thought systems [2]–[4]. Since one's emotional reactions to events are affected by the mechanisms with which information is processed, negatively biased cognitive processes can lead to maladaptive emotional and behavioral consequences [5].

Cognitive therapy for depression has been demonstrated to be an effective therapeutic modality. It aims to adjust “cognitions” rationally and/or functionally on three levels [6]. First, (Negative) Automatic thoughts emerge at a superficial level and provoke rapid, emotional cognitions. The second level involves intermediate beliefs including rules, attitudes, assumptions and strategies about internal and external events. The third level involves core beliefs that originate at the deepest levels of cognition and create longstanding and unconditional cognitive structures that can affect information processing [7]. For example, a man thinks “she doesn't care about me” when a friend of his doesn't answer his greeting. That man may hold a core belief that “I am unlovable” and may be constantly insecure about his relationships. He may assume “if I let my friends down, they will stop caring about me”. He may believe he cannot afford to have any faults. In cognitive therapy for depression, treatment begins with a discussion of the client's experience and the interaction between events, thoughts, emotions and behaviors. Next the therapist urges the client to recognize and adjust his or her automatic thoughts. Repeated evaluations of the automatic thoughts of a depressed patient reveal their biased cognitive style. Going beyond content, negatively biased thought processes are called “*cognitive distortions*” or “*thinking mistakes*” [8]. Core beliefs are considered important risk factors for developing depression [9]. Negative automatic thoughts (NAT's) are not-evidence based and do not reflect reality in functional way, they usually are produced along with cognitive distortions. For instance a NAT of “she doesn't care about me” may accompany a cognitive distortion called *personalization*. Beck et al in their seminal work outlined 7 cognitive distortions that are characteristic of depressed individuals [1]. Burns then expanded this number to 10 (See: reference [10]). Thus, detecting cognitive errors and developing insight about them is an important component of cognitive behavioral therapy for depression.

The literature includes several attempts to measure specific cognitive distortions [11]–[13]. Covin et al developed a unique two-dimension, 20-item scale to directly measure cognitive distortions [14]. Its contents and dimensions were taken directly from the work of Beck et al and Burns [1], [7], [10]. The Cognitive Distortions Scale (CDS) seeks to detect cognitive distortions held by a person providing definitions of individual cognitive distortions and illustrating them with one-paragraph case examples for each domain (the interpersonal domain and the personal achievement domain). The subject is asked to estimate how often s/he uses that ***type of thinking***. Covin et al (2011) examined the validity and reliability of the CDS in a non-clinical sample consisting of 318 undergraduates and found it a promising tool with good psychometric properties to measure cognitive distortions [14].

The primary aim of the current study was to evaluate the psychometric properties of the CDS in two Turkish samples, one clinical and one non-clinical. Second, this study aimed to examine whether the CDS's psychometric properties differed across the clinical and non-clinical samples. Third, we wanted to test the usefulness of an alternative categorical scoring system for the CDS.

## Material & Methods

### Participants

The participants totaled 325 individuals from two samples, non-clinical (sample 1) and clinically depressed (sample 2). Sample 1 consisted of 225 subjects (161 females) from various professions (e.g., medical school students, nursing students, clinical and undergraduate psychology students, and social workers) working as interns at Ankara Diskapi Yildirim Beyazit Teaching and Research Hospital. Sample 2 consisted of 100 patients diagnosed with non-psychotic major depressive disorder (MDD) (71 females), who presented to the Department of Psychiatry out-patient unit of Ankara Diskapi Y.B. Teaching and Research Hospital. Patients with psychotic disorders, bipolar disorder, mental retardation, current substance use disorder, who have less than 8 years of education, or significant cognitive decline, were excluded from the study. Healthy controls screened for active psychopathology using socio-demographic data form, and introductory interview. Healthy controls were excluded from the study if they had any diagnosable psychiatric disorder.

### Measures and Assessment Tools

#### The Cognitive Distortions Scale (CDS; Covin, Dozois, Ogniewicz, & Seeds, 2011)

This is a 20-item self-report, Likert type scale instrument developed by Covin et al in 2011 to measure 10 cognitive distortions (*mindreading, catastrophizing, all-or-nothing thinking, emotional reasoning, labeling, mental filter, overgeneralization, personalization, should statements, minimizing the positive*) using a 7-point scale (1  =  never, 7  =  all the time) [14]. Each cognitive distortion is rated in two domains: interpersonal (IP) and personal achievement (PA). In the original study, the CDS emerged as a one-factor (unitary) scale with good internal consistency (Chronbach's α = 0.85).

#### Assessment of demographic information

All participants completed a demographics and clinical information form assessing age, marital status, education, income, and employment status as well as clinical information such as substance use, suicide attempt history, physical illness, and family history of psychiatric disorders.

#### The Beck Depression Inventory (BDI; Beck, Ward, Mendelson, Mock, & Erbaugh, 1961)

This is a 21-item scale developed and revised by Beck et al. [15] and updated by Beck et al. [16] measuring emotional, cognitive, somatic and motivational symptoms. It is based on data obtained from clinical observations [17]. In the Turkish version of this test (BDI-II), a score of 17 is considered the cut off point for validity and reliability [18], [19].

#### The State Trait Anxiety Inventory (STAI; Spielberger, Gorsuch, & Lushene, 1970)

This scale was developed by Spielberger et al. [20]. The STAI consists of two sub-scales, each composed of 20-items, measuring state and trait anxiety. The STAI state sub-scale (STAI-S) asks respondents to rate how they feel ‘right now… at this moment’ using a 4-point scale (1  =  not at all, 4  =  very much so) in response to a series of self-descriptive statements. The STAI trait subscale (STAI-T) asks respondents to rate how they feel ‘in general’ using a 4-point scale (1  =  almost never, 4  =  almost always) in response to relevant statements (Chronbach's α  =  0.90). The Turkish version of the STAI has been demonstrated to be valid and reliable (Chronbach's α  =  0.94) [21].

#### Dysfunctional Attitudes Scale-Form A (DAS; Weissman and Beck, 1978)

In its original form, the DAS was developed by Weissman and Beck [22]. The 40-item short form of the DAS (Form A) was used in the current study. The DAS is a self-report questionnaire containing statements such as “It is difficult to be happy unless one is good looking, intelligent, rich and creative”. These statements are expressions of various dysfunctional attitudes frequently found in psychiatric patients and associated with vulnerability to depression. Statements are rated on a 7-point scale ranging from 1 (totally disagree with that statement) to 7 (totally agree with that statement). Its validity and reliability have been shown in many different populations [23]–[25] including the Turkish population [26]. The internal consistency of the Turkish version is good (Cronbach's α  = 0.79) and has four factors: *“perfectionism”, “need for approval”, “independent attitudes” and “mixed attitudes”*. All sub-scales except the *independent attitudes* subscale discriminated between depressive and non-depressive individuals in a Turkish population [27].

#### Automatic Thought Questionnaire (ATQ; Hollon & Kendall, 1980)

The ATQ was created by Hollon and Kendall (1980) to identify and measure the frequency of automatic thoughts associated with depression [28]. This is a 30-item self-report instrument that measures the frequency of the occurrence of negative automatic thoughts, or self-statements. Each item represents a thought and respondents rate the frequency of this thought on a 5-point scale ranging from 1 (not at all) to 5 (all the time), for the past week. The Turkish version of the ATQ has been shown to be valid and reliable with very good internal consistency (Chronbach's Alfas 0.89 to 0.91) [29].

#### The Structured Clinical Interview for DSM-IV Axis I Disorders (SCID-I; First, Spitzer, Gibbon, & Williams, 1997)

This is a structured clinical interview form developed by First et al. (1997) to diagnose DSM-IV Axis I disorders. Validity and reliability was established for the Turkish test by Özkürkçükigil, Aydemir, Yıldız, Danaca, & Köroğlu (1999) [30], [31].

### Procedures

All participants were instructed to complete the following paper-and-pencil tests. Individuals from Sample 1 filled out the measures individually in a testing room routinely used for psychological assessment. Forms and questionnaires were administered in four different orders. The socio-demographic information form and CDS were administered first for all subjects but the order of administration of the DAS, ATQ, BDI, and STAI was changed throughout the sample. In addition to this process Sample 2 subjects completed a clinical interview using The Structured Clinical Interview for DSM-IV Axis I Disorders (SCID-I). No compensation of any kind was given to the participants. All participants from Sample 1 filled out the questionnaires anonymously. However, the first 30 participants who re-took the tests two weeks later wrote down the last 4 digits of their cell phone numbers to allow pairing. A written informed consent was obtained from all participants. Since all participants including the individuals from the non-clinical group and depressed group were older than 18 years of age and not having any condition that could interfere with their capacity (e.g., psychosis, intellectual disability, or illiteracy) all the consent papers were obtained from the participants themselves and no surrogate consent procedure was conducted. This study was conducted in accord with the Helsinki Declaration and was approved by the Ethics Committee of the Diskapi Y. B. Teaching and Research Hospital.

#### Translation of the CDS

The Turkish translation of the CDS was used in the current project. When creating the Turkish version guidelines widely used in cross-cultural research were followed [32]. Accordingly, three cognitive behavioral therapists separately translated the CDS into Turkish. A consensus version was translated into back into English and reviewed a second time. The final version was then approved by the same three clinicians in addition to the authors of this paper.

#### Statistical Analyses

All analyses were conducted using SPSS for Windows. Spearman correlation coefficients were calculated for convergent validity, comparing CDS scores and subscale scores with BDI, DAS, ATQ, and STAI scores. Internal consistency was evaluated using Cronbach's α and item/total correlations (and also “if item deleted” co-efficient”) using the Spearman correlation coefficient. For parametric variables, student t tests were used to compare means. Frequencies were analyzed using a Chi-Square test. To evaluate the adequacy of the samples to construct validity analyses, Kaiser-Meyer-Olkin and Barlett tests were used. Principal component analysis with relevant rotation technique was used when evaluating the factor structure of the CDS. When determining the components of the scale, the following criteria were used: 1) Eigen values should be greater than one according to Kaiser Criterion (1961) 2) A Scree plot test was evaluated 3) all interpretations were derived from the theoretical background of the tests.

## Results

### Demographic & Clinical Properties

Sample 1 and sample 2 were similar in terms of gender (71.6% and 71% respectively were women). Sample 1 was younger and had more education (24.24±4.94 years versus 26.54±7.91 years, p<0.05; 15.56±2.2 versus 13.21±3.55 educational years, p<0.001). The two groups were made up mostly of single people (81.3% and 67%, respectively). Sample 1 had higher incomes as compared with Sample 2 (69.7% of Sample 1 had ≥ 2000 Turkish Liras whereas 45% of Sample 2 had ≥ 2000 Turkish Liras). The presence of family psychiatric history was similar in both groups (14.2% and 14% respectively).

In the clinical sample, 46% of the patients were having another DSM-IV Axis-I disorder diagnosis. MDD past episode was the commonest DSM-IV diagnosis with a proportion of 23%. It was followed by Generalized Anxiety Disorder (GAD) and Specific Phobia (SP) with 12%, Dysthymic Disorder (DD) with 11%. When the two groups (i.e., non-clinical sample and depressed individuals) were compared in relation to their levels of depression, state & trait anxiety, cognitive distortions and dysfunctional attitudes, they differed significantly on many items (please see Table 1).

**Table 1:** The means, standard deviations and comparisons of BDI, STAI, CDS, and DAS scales' scores between the clinical and non-clinical samples.

Measure	Non-clinical sample (Sample 1) N = 225		Depressive Patients (Sample 2) N = 100		P values
	Mean	SD	Mean	SD	
Beck Depression Inventory*	4.83	4.68	28.96	9.43	0.000
STAI-State Anxiety*	34.12	9.12	50.92	11.01	0.000
STAI-Trait Anxiety *	38.03	7.99	56.50	8.71	0.000
Cognitive Distortion Scale-IP*	29.92	10.70	42.70	12.36	0.000
Cognitive Distortion Scale-PA*	28.62	10.48	40.15	11.70	0.000
Cognitive Distortion Scale-Total *	58.54	20.64	82.85	22.72	0.000
DAS-Perfectionism*	44.57	15.08	60.29	19.05	0.000
DAS-Need for Approval*	38.22	10.02	49	10.91	0.000
DAS-Independent Attitudes	18.62	5.50	23.41	5.96	0.017
DAS-Mixed Attitudes	19.30	4.05	20.54	4.72	0.025

STAI = State Trait Anxiety Inventory, IP = Interpersonal subscale of CDS, PA = Personal Achievement subscale of CDS, DAS = Dysfunctional Attitudes Scale.

T tests were performed using Bonferroni correction. Accordingly p value was set p<0.005.

*Statistically significant differences between the two groups.

### Validity Measurements

#### Factor Analysis

A principal component analysis with Varimax rotation was performed for the full 20 items of the CDS. This procedure revealed four factors with eigenvalues greater than one (8.90, 1.67, 1.17, 1.05) in the Sample 1. Sixty four point three percent (64.03%) of the total variance was accounted for by four factors. CDS *catastrophizing* item of interpersonal domain (CDS-2IP), CDS *labeling* item of interpersonal domain (CDS-5IP), CDS *personalization* items of both interpersonal and personal achievement domains (CDS-8IP and CDS-8PA), CDS *should statements* items of both interpersonal and personal achievement domains (CDS-9IP and CDS9PA), CDS *minimizing or disqualifying the positive* items of interpersonal and personal achievement domains (CDS-10IP and CDS-10PA) were cross-loaded for the two different factors. Further examination of the data using Cattell's scree test and considering high rates of cross-loading indicated that it is more logical to consider these items on a unitary scale with one factor. According to this approach all items except mind reading items were loaded at a value greater than 0.10 and factor loadings were greater than 0.30 for all 10 items. On the current form 44.50% of the variance was accounted by one factor only.

The same procedure was repeated for Sample 2. This analysis revealed six factors with eigenvalues greater than one (7.91, 1.78, 1.45, 1.28, 1.15, 1.01) in Sample 2. Seventy two point ninety eight percent (72.98%) of the total variance was accounted for by those six factors. However CDS-6IP, CDS-7IP, CDS-2IP, CDS-4IP, CDS-4PA, CDS-3PA, CDS-6PA, and CDS-7PA were cross-loaded under, at least, two different factors. A Cattell's scree test consideration of very high cross-loadings indicated a one factor solution would be more reasonable for Sample 2 as well as for Sample 1. In the clinical sample factor loading was under 0.10 for six items including CDS-10PA, CDS-5IP, CDS-8PA, CDS-3PA and CDS-7PA. One factor accounted for 39.55% of the total variance.

#### Construct validity

To examine the construct validity of the CDS and its subscales, we computed correlations between CDS scores and the clinical measures included in the current study. The results of these analyses conducted both on the data from Sample 1 and Sample 2 are presented in Table 2. In addition, calculations were made for the correlations between CDS scores and scores on the DAS, its subscales, and the ATQ which measure similar cognitive tendencies in depression. These results are in Table 3. In the non-clinical group each item of the CDS was significantly correlated with BDI scores (weak to moderate correlations as indicated by correlation coefficients r = 0.158 to r = 0.338) as well as STAI-S and STAI-T scores. In the non-clinical sample every item of the CDS was correlated in some way with primary affect measures (i.e., BDI, STAI-S, and STAI-T).

**Table 2:** The correlations between the individual items of CDS and BDI, STAI-S, and STAI-T.

Individual items of CDS		Non-clinical sample (Sample 1) N = 225			Depressive Patients (Sample 2) N = 100		
		BDI	STAI-S	STAI-T	BDI	STAI-S	STAI-T
**1-Mind reading**	IP	**,192** **	**,166** *	**,253** **	,039	,080	**,331** **
	PA	**,277** **	**,273** **	**,305** **	,013	,123	,193
**2-Catastrophizing**	IP	**,268** **	**,235** **	**,384** **	,143	**,208** *	**,355** **
	PA	**,245** **	**,262** **	**,388** **	**,237** *	,113	**,284** **
**3-All-or Nothing Thinking**	IP	**,154** *	**,211** **	**,248** **	,092	,134	,118
	PA	**,218** **	**,334** **	**,327** **	−,002	,014	,022
**4-Emotional Reasoning**	IP	**,231** **	**,207** **	**,260** **	**,249** *	,085	,168
	PA	**,273** **	**,274** **	**,353** **	,062	,164	,120
**5-Labeling**	IP	,**307** **	**,165** *	**,333** **	,052	,058	,120
	PA	**,274** **	**,262** **	**,386** **	,069	,087	,176
**6-Mental Filter**	IP	**,298** **	**,214** **	**,398** **	,033	,086	**,240** *
	PA	**,247** **	**,224** **	**,348** **	,102	,135	,159
**7-Overgenaralization**	IP	**,329** **	**,275** **	**,441** **	**,266** **	**,201** *	**,293** **
	PA	**,329** **	**,275** **	**,441** **	,171	,095	,152
**8-Personalization**	IP	**,175** **	**,213** **	**,337** **	,125	,064	**,237** *
	PA	**,220** **	**,356** **	**,203** **	,127	,071	**,209** *
**9-Should statements**	IP	,**279** **	**,363** **	**,201** **	,075	,157	**,341** **
	PA	**,338** **	**,385** **	**,243** **	,041	,096	**,216** *
**10-Minimizing or Disqualifying the Positive**	IP	,**228** **	**,400** **	**,237** **	**,267** **	,179	**,322** **
	PA	**,219** **	**,240** **	**,420** **	,152	−,030	,130

Pearson correlation coefficients were calculated between the variables.

*Statistically significant at the level of p<0.05.

**Statistically significant at the level of p<0.001.

**Table 3:** Correlations between CDS scores and DAS & ATQ scores in two samples.

	Non-clinical sample (Sample 1) N = 225					Depressive Patients (Sample 2) N = 100				
	DAS-P	DAS-NA	DAS-IA	DAS-MA	ATQ	DAS-P	DAS-NA	DAS-IA	DAS-MA	ATQ
**Cognitive Distortion Scale-IP**	,380**	,433**	,269**	,209**	.371**	,324**	,239*	,132	,130	,391**
**Cognitive Distortion Scale-PA**	,399**	,424**	,272**	,177**	,404**	,364**	,262**	,170	,183	,305**
**Cognitive Distortion Scale-Total**	,400**	,440**	,278**	,198**	,397**	,364**	,265**	,160	,165	,370**

DAS-P: Dysfunctional Attitude Scale-Perfectionism subscale; DAS-NA: Dysfunctional Attitude Scale-Need for Approval subscale; DAS-IA: Dysfunctional Attitude Scale-Independent Attitudes subscale; DAS-MA: Dysfunctional Attitude Scale-Mixed Attitudes Subscale. ATQ: Automatic Thought Questionnaire. IP = Interpersonal subscale of CDS, PA = Personal Achievement subscale of CDS.

Pearson correlation coefficients were calculated between the variables.

*Statistically significant at the level of p<0.05.

**Statistically significant at the level of p<0.001.

However, in the clinical sample only *catastrophizing* (personal achievement domain), *emotional reasoning* (interpersonal domain), *overgeneralization* (interpersonal domain), *and disqualifying the positive* (interpersonal domain) were significantly correlated with total BDI scores. In the clinical group STAI-T scores were correlated to ten CDS items whereas BDI scores were correlated to only four items of the CDS (See Table 2).

One of the questions raised by the original study was whether there was a difference between errors made in interpersonal contexts and achievement contexts. Since the intention of creating a two-domain scale (e.g., interpersonal context and achievement context) was to test if some thinking errors occur in one context rather than in the other. Ten dependent *t* tests were conducted for each thinking mistake with a corrected p value of p<0.01. Both in the non-clinical sample and the clinical sample *mind-reading* (p = 0.001; p = 0.009), *emotional reasoning* (p = 0.00; p = 0.001), and *overgeneralization* (p = 0.00; p = 0.001) occurred more frequently in the interpersonal domain.

#### Discriminant Validity

Table 1 shows the mean scores of the CDS and its subscales in the clinical and non-clinical samples. The CDS and its subscales' scores were significantly statistically different between the two groups.

### Reliability Measurements

Internal consistency was computed using Cronbach's α. For the sample 1, Cronbach's α values were 0.933 for the CDS total, 0.871 for the interpersonal subscale, 0.874 for the personal achievement subscale. Item total score correlations varied 0.495 to 0.711. No significant change was observed if any item was deleted out of the 20 items. For the sample 2, Cronbach's α values were 0.918 for the CDS total, 0.868 for the interpersonal subscale, 0.847 for the personal achievement subscale. Item total score correlations varied from 0.478 to 0.703 for the 20-item CDS. Once again, no significant change was observed if any item was deleted out of the 20 items in sample 2. To assess Test-retest Reliability, 30 individuals from Sample 1 were re-administered the CDS two weeks later. CDS score totals were strongly correlated between the two assessment times (r = 0.783, p<0.001). The interpersonal and the individual achievement subscale scores showed similar correlations (r = 0.735, p<0.001 and r = 0.809, p<0.001).

### Results for the CDS using alternative scoring procedure

Although no other scoring system was offered in the original paper, raw scores were used in previous statistics as recommended in the original study. However, an alternative scoring procedure was also used. In this procedure an item was rated as “1-exists” if a participant marked 6 or 7 on the Likert type scale and “0-doesn't exist” if a participant marked below the 6. A similar approach was used to score the Young Schema Questionnaire, developed for assessing early maladaptive schemas (see: Young, Klosko, & Weishaar, 2003, p. 75) [33]. Every participants' number of cognitive distortions were calculated. The number of cognitive distortions endorsed by each individual participant showed a difference between the two groups (p<0.001). In the non-clinical sample the mean number of cognitive distortion was 1.66±2.87 whereas for the clinical sample the mean was 5.69±4.65. The total numbers of cognitive distortions were significantly correlated with STAI-state, STAI-trait, and BDI scores in non-clinical (r = 0.233, p<0.01; r = 0.418, p<0.01; r = 0.326, p<0.01 respectively) and clinical samples (r = 0.187, p>0.05; r = 0.343, p<0.01; r = 0.299, p<0.01 respectively).

## Conclusions

The aim of the current study was twofold. First, to examine the psychometric properties, reliability, and validity of the CDS. Second, to test an alternative scoring method evaluating cognitive distortions as categorical entities (exists or doesn't exist). The Psychometric properties that appeared in the current study were mostly similar to the results of the original study. We suggest that the CDS is a valid and reliable measure of cognitive distortions in both clinical and non-clinical samples with some caution about its use for clinical and experimental purposes. In addition, a categorical approach in scoring the CDS appeared more relevant especially in the clinical sample.

The two-factor structure was not supported in this study, in accord with to the original study that created the CDS [14]. This was the case for both clinical and non-clinical groups, again in accord with the previous studies [14], [34]. Although our factor analytic work supports a one-factor model it doesn't necessarily imply that a two-factor model is clinically not useful. In the current study cognitive distortions including mind-reading, emotional reasoning and overgeneralization consistently appeared more frequently in the interpersonal domain. Again this is partially in accord with Covin et al.'s study. In addition, they found that *mental filtering* was reported more frequently in one of the two non-clinical samples of the study. These findings suggest that CDS scores endorsed in interpersonal and personal achievement contexts may not be related to given cognitive errors, but rather may be related to an individual's deeper cognitive structures, like assumptions or core beliefs [5]. Since the CDS items are based on two different vignettes for each cognitive distortion it might give us more opportunities to find cognitive distortions in two main contexts (i.e., interpersonal and personal achievement) [35]. Accordingly, using the CDS as it is has a potential in clinical settings despite the lack of statistical evidence in this regard.

Validity findings along with very good reliability coefficients across the two samples indicate that the CDS is a valid and reliable measure in clinical and non-clinical groups. The CDS also differentiated the depressive and non-depressive groups.

Despite good internal consistency and validity findings some unexpected results appeared. In the non-clinical sample, like in the study of Covin et al., each item of the CDS was correlated to depression, and anxiety scores. The correlations between the trait anxiety measures and cognitive distortions were usually stronger than those between the depression scores and cognitive distortions' scores. In the non-clinical sample negative affective states were generally concurrent with cognitive distortions. However, in the clinical (depressed) group some cognitive distortions (i.e., catastrophizing, emotional reasoning, overgeneralization, and Minimizing or Disqualifying the Positive) had more concurrent predictive values for depressive affect while some cognitive distortions (i.e., mind reading, catastrophizing, mental filter, overgeneralization, personalization, should statements, and Minimizing or Disqualifying the Positive) had more concurrent predictive values for trait anxiety features. It is very interesting that cognitive errors had stronger correlations for the trait anxiety measure. This finding when combined with those from Covin et al.'s study suggests that cognitive distortions are not specific to depressive symptoms but rather related to general distress or negative affect.

When we look at the correlations between the CDS and the scores of the measures that assess similar but not identical cognitive functions (i.e., dysfunctional attitudes and negative automatic thoughts) we observe stronger correlations. However, correlation analyses does not support the two-dimension structure of the CDS since DAS-NA scores had stronger correlations to CDS total and subscales scores in the non-clinical group whereas the DAS-P scores had stronger correlations to the same scores. Negative automatic thoughts showed similar correlations across the two samples and to CDS, CDS-PA, CDS-IP scale scores. These findings support that the cognitive distortions are more related to automatic thoughts and dysfunctional attitudes than to depressive symptoms.

In addition to psychometric analyses of the CDS we assessed the usefulness of an alternative approach in scoring CDS in this study. Since cognitive distortions seen in depression rarely appear individually but instead appear concurrently with one another [1], [36] we rated each cognitive distortion item either to exist or not to exist and counted the number of cognitive distortions endorsed. The number of cognitive distortions distinguished the depressive and non-depressive groups. In addition, those scores were correlated more strongly to depressive symptom severity while correlations between the cognitive distortions and trait anxiety scores were similar. These findings suggest that a categorical approach to cognitive distortions may be more relevant to understanding the relationship between cognitive distortions and severity of depression.

An obvious limitation of this study is that the individuals in clinical sample filled out the questionnaires when they were depressed. Since current mood effects the endorsement of dysfunctional beliefs [37] clinical data should be considered with caution. The CDS has the same limitations as other self-report measures. A “perceived bias” cannot be ruled out due to the absence of a structured attempt to measure the actual demonstration of errors. Finally, these results cannot be generalized to all clinical and non-clinical populations because being a volunteer in a study reflects special attitudes linked to self selection biases.

In sum, the CDS is a valid and reliable scale measuring cognitive distortions effectively in clinical and non-clinical populations. Although its subscales may be used in clinical settings it seems that a unitary scale could measure cognitive distortions in interpersonal and personal achievement contexts. Further, a categorical scoring of the scale may be more useful in clinical settings.
